# Supplementary material for: “Should I Say Something?”: A Simulation Curriculum on Addressing Lapses in Professionalism to Improve Patient Safety
Source: MedEdPORTAL. 2023 Dec 12;19:11359. doi: 10.15766/mep_2374-8265.11359 (PMC10713868; doi:10.15766/mep_2374-8265.11359)
Supplement: Supplementary file 1 — Case Summary.docxNarrated Preclass Presentation.m4vCharacter Role Cards.docxFlowchart for Simulation Role-Play.pdfBrief and Debrief Guide.docxCritical Actions Checklist.docxSISS Pre- and Postsurveys.docx [file mep_2374-8265.11359-s001.zip › C. Character Role Cards.docx]

**Appendix C: Role Play Character Cards**

Each participant except the Patient should read their character description to the group in the briefing. Participants should share their Patient Care Information if asked during the role play. The participant playing the role of Patient shares their character description in the debriefing.

It is helpful to have several sets of laminated copies of each card. Make sure to have one complete set for each group of learners.

**=============================================================================================**

**Role: Attending**

You are an experienced Surgeon with a busy, successful practice. You are known as friendly and likable to your colleagues, and you get along well with the residents. You have been teaching in the Surgery Clerkship for many years and consider yourself a good teacher, although your teaching is limited to occasional lectures and teaching in the OR. You prefer teaching residents over students. You are a 4^th^ generation, life-long resident of This Great City. You have 4 children and a stay-at-home spouse. You are under a great deal of financial pressure with a mortgage, college tuitions, and elderly parents. You would like to go up for academic promotion but have not been as productive as needed to advance. Your department is under financial duress and your clinical hours were recently increased. You have 4 surgeries scheduled for today.

Patient care information: You received phone sign-out from the overnight team. The intern on call evaluated the patient. The overnight attending did not see the patient and signed out to you “you just need to lay eyes on them and send them home”.

**=============================================================================================**

**Role: Patient (this role may be played by non-learner)**

You are a 38 yo immigrant from a non-English speaking country and have 12^th^ grade education. You understand some English but do not feel comfortable speaking. You are in the US legally and work full-time at a job that pays $15/hour, but you do not qualify for Medicaid and have no health insurance. You have 4 children and your spouse, who is undocumented, recently lost a job; you are now the sole provider for your family. You send money back to your parents every month. You developed abdominal pain, nausea, and fever last night, and were unable to go to work this morning when you started vomiting. You came to the Emergency Department with your oldest child, a son who is 17 years old and in high school. The pain is getting worse, and you feel like you are getting a fever. The pain medication given by the nurse is not helping. You are worried this is serious but also worried about the cost of being treated.

Patient care information: You respond yes to most questions that you are asked to be agreeable. You are visibly uncomfortable with abdominal pain, and you wince in response to the mimed abdominal examination. When it becomes clear the recommendation is for hospitalization and surgery, you inform the team you must leave because of work, family obligations and concerns over the cost.

Additional Instructions: Some team members speak or understand your language and may attempt to converse with you. If you both speak another language you may converse in that, or you may premise your communication with those team members by stating “In our language….” before conversing.

You may attempt to ask for an interpreter if you like, or you can try to communicate without the interpreter. Creativity is welcome!

NOTE: This role requires you to lay down on the stretcher and to pretend the Attending is examining your abdomen. **There will be NO actual physical touch or contact, and you do NOT need to expose any body part. If you prefer NOT to play this role, inform the facilitator and you will be re-assigned.**

**=============================================================================================**

**Role: Resident**

You are a senior resident on the Surgery service. You had planned to be on a research elective but were asked to cover the service for a few months while 2 residents are out on medical leave. You have had difficulty with adjusting to being back on the wards, as your research is at a critical point. You enjoyed teaching but since coming back to clinical medicine find that the students are less excited about being on the Surgery Clerkship, and the interns seem unprepared. The Attending is well-connected and on the fellowship selection committee for the fellowship you wish to enter. You are not married and live with a roommate; your family lives in another state and you are not close to them.

There are 7 other patients on the service, and 3 of today’s surgical patients will be admitted. You have two consults on in-patients today.

Patient care information: Your co-resident admitted the patient last night and signed out as “probably ruling out for appendicitis”. However, the patient is now febrile and looks unwell.

Additional Instructions: Some team members speak or understand the patient’s language and may attempt to converse with them. They will state “In our language….” before conversing, indicating that you should NOT understand that conversation.

Creativity is welcome!

**=============================================================================================**

**Role: Intern**

You are a preliminary intern in the Surgery residency program. You were unsure what surgical specialty you wanted to enter, but you admire the Attending and now wish to stay in this residency. You love teaching medical students and hope to one day be on staff in This Great Hospital. You are very close to your large family and are engaged to be married in 5 weeks. You feel lucky to be at This Great Hospital but worry about paying back your student loans. Your grandparents are originally from the same country as the Patient, and you speak the same language but don’t know any medical terms. There are 7 other patients on the service, and 3 of today’s surgical patients will be admitted. You have not returned several pages from nurses regarding IV fluid rates, foley catheter removal, and discharge planning.

Patient care information: You pre-rounded on this patient with the student this morning, and you think the exam showed rebound and guarding. You are not sure if the abdominal exam is an “acute abdomen”. You had received sign-out from the night float intern that the patient was getting better.

Additional Instructions: You and the colleague playing the Patient role can speak to and understand one another and may attempt to converse. Others may also speak or understand the language. IF you both speak another language you may converse in that, or you may premise your communication with those team members or the Patient by stating “In our language….” before conversing.

Creativity is welcome!

**=============================================================================================**

**Role: Clerkship Student**

You are a clerkship student on your first clerkship, Surgery. You came to medical school planning to enter primary care, but since starting Surgery, you have been strongly considering this as a career. You have just completed one of the online training modules about using interpreter services, and you know every floor has a dedicated portable “interpreter phone” at the nurses’ station. While you do not speak the same language as the Patient, your family are immigrants as well, and you have helped several relatives with navigating the medical system. You want to do your best for the patient, but you know the team must get to the OR on time; yesterday, you witnessed a different Attending yelling at the Resident when the first case was 5 minutes late in starting. Today is the first day you will be presenting a patient on rounds, and you want to make a good impression.

Patient care information: You pre-rounded on this patient with the intern this morning (your first experience of pre-rounds). You noted the patient is febrile to 101.2, has an absence of bowel sounds, and there is rebound and guarding on abdominal exam.

**=============================================================================================**

**Role: Student Sub-Intern (this role may be omitted if only 4 learners)**

You are a student on your sub-internship. You are the 3^rd^ member of your family to attend This Great Medical School, and you plan a career in Orthopedics. You feel you must do well on this clerkship and need to impress the Attending, who knows your parents socially. You have traveled extensively and spent 8 months in the country the Patient is from working in a clinic and consider yourself fluent in the language. You have applied to work in the same research lab as the Resident, and the Resident has mentioned you would be a great fit for the lab. You plan to stay at This Great Hospital for residency.

Patient care information: You have not seen this patient today and have no information for the team.

Additional Instructions: You and the colleague playing the Patient role can speak to and understand one another and may attempt to converse. Others may also speak or understand the language. IF you both do speak another language, you may converse in that, or you may premise your communication with those team members or the Patient by stating “In our language….” before conversing.

Creativity is welcome!
