# Supplementary material for: “Should I Say Something?”: A Simulation Curriculum on Addressing Lapses in Professionalism to Improve Patient Safety
Source: MedEdPORTAL. 2023 Dec 12;19:11359. doi: 10.15766/mep_2374-8265.11359 (PMC10713868; doi:10.15766/mep_2374-8265.11359)
Supplement: Supplementary file 1 — Case Summary.docxNarrated Preclass Presentation.m4vCharacter Role Cards.docxFlowchart for Simulation Role-Play.pdfBrief and Debrief Guide.docxCritical Actions Checklist.docxSISS Pre- and Postsurveys.docx [file mep_2374-8265.11359-s001.zip › D. Flowchart for Simulation Role-Play.pdf]

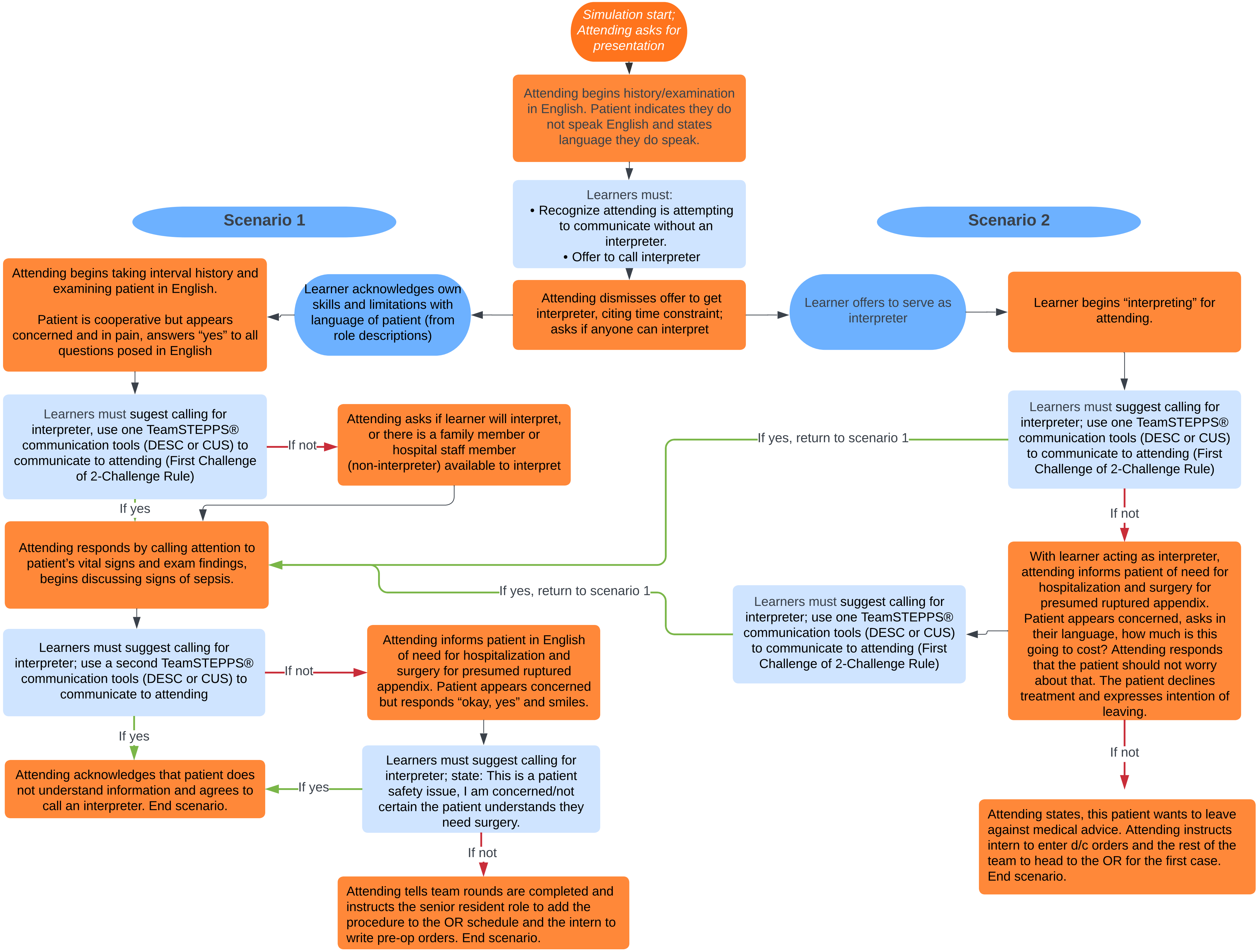
Simulation start; Attending asks for presentation
Attending begins history/examination in English. Patient indicates they do not speak English and states language they do speak.
Learners must:
• Recognize attending is attempting to communicate without an interpreter.
• Offer to call interpreter
Attending dismisses offer to get interpreter, citing time constraint; asks if anyone can interpret
Scenario 1
Learner acknowledges own skills and limitations with language of patient (from role descriptions)
Attending begins taking interval history and examining patient in English.
Patient is cooperative but appears concerned and in pain, answers "yes" to all questions posed in English
Learners must suggest calling for interpreter, use one TeamSTEPPS® communication tools (DESC or CUS) to communicate to attending (First Challenge of 2-Challenge Rule)
If not
Attending asks if learner will interpret, or there is a family member or hospital staff member (non-interpreter) available to interpret
If yes
Attending responds by calling attention to patient's vital signs and exam findings, begins discussing signs of sepsis.
Learners must suggest calling for interpreter; use a second TeamSTEPPS® communication tools (DESC or CUS) to communicate to attending
If not
Attending informs patient in English of need for hospitalization and surgery for presumed ruptured appendix. Patient appears concerned but responds "okay, yes" and smiles.
If yes
Attending acknowledges that patient does not understand information and agrees to call an interpreter. End scenario.
If yes
Learners must suggest calling for interpreter; state: This is a patient safety issue, I am concerned/not certain the patient understands they need surgery.
If not
Attending tells team rounds are completed and instructs the senior resident role to add the procedure to the OR schedule and the intern to write pre-op orders. End scenario.
Scenario 2
Learner offers to serve as interpreter
Learner begins "interpreting" for attending.
Learners must suggest calling for interpreter; use one TeamSTEPPS® communication tools (DESC or CUS) to communicate to attending (First Challenge of 2-Challenge Rule)
If yes, return to scenario 1
If not
With learner acting as interpreter, attending informs patient of need for hospitalization and surgery for presumed ruptured appendix. Patient appears concerned, asks in their language, how much is this going to cost? Attending responds that the patient should not worry about that. The patient declines treatment and expresses intention of leaving.
Learners must suggest calling for interpreter; use one TeamSTEPPS® communication tools (DESC or CUS) to communicate to attending (First Challenge of 2-Challenge Rule)
If yes, return to scenario 1
If not
Attending states, this patient wants to leave against medical advice. Attending instructs intern to enter d/c orders and the rest of the team to head to the OR for the first case. End scenario.
