# Supplementary material for: “Should I Say Something?”: A Simulation Curriculum on Addressing Lapses in Professionalism to Improve Patient Safety
Source: MedEdPORTAL. 2023 Dec 12;19:11359. doi: 10.15766/mep_2374-8265.11359 (PMC10713868; doi:10.15766/mep_2374-8265.11359)
Supplement: Supplementary file 1 — Case Summary.docxNarrated Preclass Presentation.m4vCharacter Role Cards.docxFlowchart for Simulation Role-Play.pdfBrief and Debrief Guide.docxCritical Actions Checklist.docxSISS Pre- and Postsurveys.docx [file mep_2374-8265.11359-s001.zip › G. SISS Pre- and Postsurveys.docx]

**G. Medical Professionalism: Pre-Session Self-Assessment Questionnaire**

The clerkship year is designed to be the first immersive step in your journey toward becoming a practicing medical professional. You will assist in patient care on teams with faculty and resident physicians who are formally entrusted both with your clinical training and performance assessment. As the concept of “professionalism” will feature prominently in your training and assessment, it is very important that you have working knowledge of what it entails in the clinical setting.

**Last four digits of cell phone #: ________

*(Note: All responses are confidential and de-identified. These responses will be used solely to pair pre- and post- surveys.)*

Age (years): ___

Gender (circle): M F other

**Please use this scale to rate your level of agreement with the following statements regarding your understanding of medical professionalism:**

| 1 | 2 | 3 | 4 | 5 |
| --- | --- | --- | --- | --- |
| Strongly Disagree | Disagree | Neutral | Agree | Strongly Agree |

**Circle your answer for each item below. Please note “trainees” includes Residents, Fellows and Interns.**

| I am familiar with the behaviors that constitute medical professionalism. | | | 1 2 3 4 5 | | | |
| --- | --- | --- | --- | --- | --- | --- |
| Lack of professionalism among **medical students** adversely effects the learning environment on the wards. | | | 1 2 3 4 5 | | | |
| Lack of professionalism among **attendings** adversely effects the learning environment on the wards. | | | 1 2 3 4 5 | | | |
| Lack of professionalism among **trainees** adversely effects the learning environment on the wards. | | | 1 2 3 4 5 | | | |
| Lack of professionalism among **nurses** **or** **other** **personnel** adversely effects the learning environment on the wards. | | | 1 2 3 4 5 | | | |
| I encounter lapses in professional behavior by **attendings** frequently. | | | 1 2 3 4 5 | | | |
| I encounter lapses in professional behavior by **trainees** frequently. | | | 1 2 3 4 5 | | | |
| I encounter lapses in professional behavior by **students** frequently. | | | 1 2 3 4 5 | | | |
| I encounter lapses in professional behavior by **nurses or other personnel** frequently. | | | 1 2 3 4 5 | | | |
| I encounter inappropriate behavior by **patients** frequently. | | | 1 2 3 4 5 | | | |
| Faculty and trainees factor medical student “professionalism” into grades and assessments. | | | 1 2 3 4 5 | | | |
| I am aware of how faculty and trainees assess professionalism among medical students. | | | 1 2 3 4 5 | | | |
| It is professionally expected to sacrifice a healthy work-life balance to do well in clinical rotations. | | | 1 2 3 4 5 | | | |
| I know how to communicate with team members when I have a concern about patient safety in a medical team setting. | | | 1 2 3 4 5 | | | |
|  | | |  | | | |
| I feel comfortable communicating with team members when I have a concern about patient safety in a medical team setting. | | | 1 2 3 4 5 | | | |
| I feel empowered, as a medical student, to address lapses of professionalism in other **students**. | | | 1 2 3 4 5 | | | |
| I feel empowered, as a medical student, to address lapses of professionalism in **trainees**. | | | 1 2 3 4 5 | | | |
| I feel empowered, as a medical student, to address lapses of professionalism in **attending** **physicians**. | | | 1 2 3 4 5 | | | |
| I feel empowered, as a medical student, to address inappropriate behavior by **patients**. | | | 1 2 3 4 5 | | | |
| I can recognize and identify potential solutions to common ethical dilemmas in the PCE. | | | 1 2 3 4 5 | | | |
| Medical students receive adequate training to act in a professional manner. | | | 1 2 3 4 5 | | | |
| My medical education has prepared me well to address lapses of professionalism I may witness. | | | 1 2 3 4 5 | | | |
| **Please rank the following as to importance to professionalism** | **Not Important** | **Somewhat Important** | | **Neutral** | **Important** | **Extremely Important** |
| Working well with other physicians |  |  | |  |  |  |
| Working well on a medical team with other professions |  |  | |  |  |  |
| Punctuality |  |  | |  |  |  |
| Appearance |  |  | |  |  |  |
| Completion of Administrative Duties |  |  | |  |  |  |
| Honesty |  |  | |  |  |  |
| Compassion |  |  | |  |  |  |
| Respect for others |  |  | |  |  |  |
| Patience |  |  | |  |  |  |
| Communication |  |  | |  |  |  |
| Leadership |  |  | |  |  |  |

**Have you witnessed concerns of professionalism in medical school?**

**If you feel comfortable doing so, please describe the incident.**

**How did you react to the concerns about professionalism?**

**talk with friends**

**talk with advisor or other faculty member**

**talk with administration**

**speak directly to person involved**

**other response**

**Medical Professionalism: Immediate Post-Session Self-Assessment Questionnaire**

The clerkship year is designed to be the first immersive step in your journey toward becoming a practicing medical professional. You will assist in patient care on teams with faculty and resident physicians who are formally entrusted both with your clinical training and performance assessment. As the concept of “professionalism” will feature prominently in your training and assessment, it is very important that you have working knowledge of what it entails in the clinical setting.

**Last four digits of cell phone #: ________

*(Note: All responses are confidential and de-identified. These responses will be used solely to pair pre- and post- surveys.)*

Age (years): ___

Gender (circle): M F other

**Please use this scale to rate your level of agreement with the following statements regarding your understanding of medical professionalism:**

| 1 | 2 | 3 | 4 | 5 |
| --- | --- | --- | --- | --- |
| Strongly Disagree | Disagree | Neutral | Agree | Strongly Agree |

**Circle your answer for each item below. Please note “trainees” includes Residents, Fellows and Interns.**

| I am familiar with the behaviors that constitute medical professionalism. | | | 1 2 3 4 5 | | | |
| --- | --- | --- | --- | --- | --- | --- |
| Lack of professionalism among **medical students** adversely effects the learning environment on the wards. | | | 1 2 3 4 5 | | | |
| Lack of professionalism among **attendings** adversely effects the learning environment on the wards. | | | 1 2 3 4 5 | | | |
| Lack of professionalism among **trainees** adversely effects the learning environment on the wards. | | | 1 2 3 4 5 | | | |
| Lack of professionalism among **nurses** **or** **other** **personnel** adversely effects the learning environment on the wards. | | | 1 2 3 4 5 | | | |
| I encounter lapses in professional behavior by **attendings** frequently. | | | 1 2 3 4 5 | | | |
| I encounter lapses in professional behavior by **trainees** frequently. | | | 1 2 3 4 5 | | | |
| I encounter lapses in professional behavior by **students** frequently. | | | 1 2 3 4 5 | | | |
| I encounter lapses in professional behavior by **nurses or other personnel** frequently. | | | 1 2 3 4 5 | | | |
| I encounter inappropriate behavior by **patients** frequently. | | | 1 2 3 4 5 | | | |
| Faculty and trainees factor medical student “professionalism” into grades and assessments. | | | 1 2 3 4 5 | | | |
| I am aware of how faculty and trainees assess professionalism among medical students. | | | 1 2 3 4 5 | | | |
| It is professionally expected to sacrifice a healthy work-life balance to do well in clinical rotations. | | | 1 2 3 4 5 | | | |
| I know how to communicate with team members when I have a concern about patient safety in a medical team setting. | | | 1 2 3 4 5 | | | |
|  | | |  | | | |
| I feel comfortable communicating with team members when I have a concern about patient safety in a medical team setting. | | | 1 2 3 4 5 | | | |
| I feel empowered, as a medical student, to address lapses of professionalism in other **students**. | | | 1 2 3 4 5 | | | |
| I feel empowered, as a medical student, to address lapses of professionalism in **trainees**. | | | 1 2 3 4 5 | | | |
| I feel empowered, as a medical student, to address lapses of professionalism in **attending** **physicians**. | | | 1 2 3 4 5 | | | |
| I feel empowered, as a medical student, to address inappropriate behavior by **patients**. | | | 1 2 3 4 5 | | | |
| I can recognize and identify potential solutions to common ethical dilemmas in the PCE. | | | 1 2 3 4 5 | | | |
| Medical students receive adequate training to act in a professional manner. | | | 1 2 3 4 5 | | | |
| My medical education has prepared me well to address lapses of professionalism I may witness. | | | 1 2 3 4 5 | | | |
| **Please rank the following as to importance to professionalism** | **Not Important** | **Somewhat Important** | | **Neutral** | **Important** | **Extremely Important** |
| Working well with other physicians |  |  | |  |  |  |
| Working well on a medical team with other professions |  |  | |  |  |  |
| Punctuality |  |  | |  |  |  |
| Appearance |  |  | |  |  |  |
| Completion of Administrative Duties |  |  | |  |  |  |
| Honesty |  |  | |  |  |  |
| Compassion |  |  | |  |  |  |
| Respect for others |  |  | |  |  |  |
| Patience |  |  | |  |  |  |
| Communication |  |  | |  |  |  |
| Leadership |  |  | |  |  |  |

**Medical Professionalism: Six Month Post-Session Self-Assessment Questionnaire**

Age (years): ___

Sex (circle): M F other

Clerkships I have completed so far (including current): _________________________________________________________________________________

Please use this scale to rate your level of agreement with the following statements regarding your understanding of medical professionalism:

| 1 | 2 | 3 | 4 | 5 |
| --- | --- | --- | --- | --- |
| Strongly Disagree | Disagree | Neutral | Agree | Strongly Agree |

Circle your answer for each item below.

|  | Strongly  Disagree | Strongly  Agree |
| --- | --- | --- |
| 1. I am familiar with the behaviors that constitute medical professionalism. | 1 2 3 4 5 | |
| 2. Lack of professionalism is an issue on the medical wards. | 1 2 3 4 5 | |
| 3. Faculty and trainees factor medical student “professionalism” into grades and evaluations. | 1 2 3 4 5 | |
| 4. I am aware of how faculty and trainees assess professionalism among medical students. | 1 2 3 4 5 | |
| 5. It is professionally acceptable to sacrifice a healthy work-life balance to do well in clinical rotations. | 1 2 3 4 5 | |
| 6. I know how to proceed when I have a concern about patient safety in a medical team setting. | 1 2 3 4 5 | |
| 7. I feel empowered, as a medical student, to address lack of professionalism in my peers (co-medical students). | 1 2 3 4 5 | |
| 8. I feel empowered, as a medical student, to address lack of professionalism in my superiors (trainees, attending physicians). | 1 2 3 4 5 | |
| 9. I can recognize and identify potential solutions to common ethical dilemmas in the PCE. | 1 2 3 4 5 | |
| 10. Overall, I feel well prepared in the area of professionalism. | 1 2 3 4 5 | |
| Lastly, based on your experiences on the wards this year: | | |

| 11. Faculty/trainees have been unprofessional towards patients. | 1 2 3 4 5 |
| --- | --- |
| 12. Faculty/trainees have been unprofessional towards me. | 1 2 3 4 5 |
| 13. I have seen medical students demonstrate unprofessional behaviors on the wards. | 1 2 3 4 5 |

Examples of unprofessional behavior(s) that I have seen on the wards include:

___________________________________________________________________________________________________________________________________________________________________________________________________________________________________________________________

**Last four digits of cell phone #: ________

(Note: All responses are confidential and de-identified. These responses will be used solely to pair pre- and post- surveys.)
