# Supplementary material for: “Should I Say Something?”: A Simulation Curriculum on Addressing Lapses in Professionalism to Improve Patient Safety
Source: MedEdPORTAL. 2023 Dec 12;19:11359. doi: 10.15766/mep_2374-8265.11359 (PMC10713868; doi:10.15766/mep_2374-8265.11359)
Supplement: Supplementary file 1 — Case Summary.docxNarrated Preclass Presentation.m4vCharacter Role Cards.docxFlowchart for Simulation Role-Play.pdfBrief and Debrief Guide.docxCritical Actions Checklist.docxSISS Pre- and Postsurveys.docx [file mep_2374-8265.11359-s001.zip › A. Case Summary.docx]

| **Appendix A: Simulation Case**  **SIMULATION CASE TITLE: “Should I say something?”: A Simulation Curriculum on Addressing Lapses in Professionalism to Improve Patient Safety**  **AUTHORS: Flier, Richards, Hacker, Hovaguimian, Sullivan, Vanka, Royce**  **LEARNER AUDIENCE: Undergraduate medical learners** | |
| --- | --- |
| **PATIENT NAME: Patient**  **PATIENT AGE: 38**  **CHIEF COMPLAINT: nausea, vomiting, abdominal pain**  **PHYSICAL SETTING: hospital room with bed, can be in-patient, observation or emergency department.** | |
| **Brief narrative description of case** | *Include the presenting patient chief complaint and overall learner goals for this case*  The patient is a 38yo person who presented to the Emergency Department around 4AM with progressive nausea, vomiting, and abdominal pain. The pain began suddenly, was initially generalized, but settled in the right lower quadrant, and was rated as a 9/10. The patient came into the ED after work, accompanied by their 16yo son. The patient has limited English proficiency and requires an interpreter. The overnight acute care surgery (ACS) intern evaluated the patient and kept the patient for observation. The patient seemed to improve, and the intern signs out the patient to the day team as needing to be seen by the team prior to discharging the patient. The patient however has become more symptomatic, with signs of sepsis and acute abdomen.  The patient was afebrile on presentation, with a tender abdomen, no CVAT, rebound or guarding, but had an elevated WBC (12.6K). There were no other significant lab abnormalities. The overnight team was initially concerned for appendicitis and ordered a CT of the abdomen and pelvis; however this was initially read as negative for appendicitis, and the overnight team signed out to the day team that the patient was ready for discharge.  The surgical team rounds on the patient at 7:20AM and discovers the patient’s condition has deteriorated, with signs of acute abdomen, tachycardia, and hypotension. The attending recommends urgent exploratory surgery for suspected appendicitis. The attending does not acknowledge a language barrier but realizes the worsening clinical situation. The attending tries to communicate to the patient without using an interpreter. When confronted by team members, the attending acknowledges the language barrier, but perseveres, citing time limitations. The team members work together to redirect the attending to accept the need for an interpreter.  **Goals for learners:**  1. Recognize the lapse in professionalism  2. Apply communication tools from TeamSTEPPS® to address conflict within the team  3. Understand the role poor communication plays in medical error |
| **Primary Learning Objectives** | *What should the learners gain in terms of knowledge and skill from this case? Use action verbs and utilize Bloom’s Taxonomy as a conceptual guide*  1. List 5 key components of professional behavior related to patient safety.  2. Define the three TeamSTEPPS® communication tools (“CUS” words, Two-Challenge Rule, DESC-ribe).  3. Describe 1 challenge and 1 benefit to speaking up about lapses in professional behavior related to patient safety.  4. Employ TeamSTEPPS® communication tools to communicate within a medical team. |
| **Critical Actions** | *List which steps the participants should take to successfully manage the simulated patient. These should be listed as concrete actions that are distinct from the overall learning objectives of the case.*  1. Recognize the patient has Limited English Proficiency and needs interpreter services.  2. Recognize the Attending is exhibiting a lapse in professional behavior.  3. Offer to call for interpreter.  4. Use C-U-S or DESC-ribe strategy to communicate with Attending.  5. Use the 2-Challenge Rule.  6. Support other members of the team in communication attempts.  7. Successfully call for Interpreter. |
| **Learner Preparation or Prework** | *What information should the learners be given prior to initiation of the case?*  Learners should review the pre-recorded narrated slide set prior to the simulation. They should be aware of the time limitation of 7 minutes to complete the role play. They should be aware they are expected to identify a lapse in professional behavior, and that they will need to intervene to ensure patient safety. They should be aware there are 3-4 other learners in the role play, and the learners are expected to function as a team to communicate a shared goal to the team member exhibiting the lapse in professional behavior. |

| **Initial Presentation** | | | | |
| --- | --- | --- | --- | --- |
| **Initial vital signs** | T 38.2°C, BP 90/60, HR 110, RR 22, SpO2 99% 2L NC | | | |
| **Overall Appearance** | The room contains a stretcher or hospital bed and a monitor displaying vital signs. The monitor may be a paper chart, or an electronic monitor.  The Patient is on the bed/stretcher in obvious discomfort from abdominal pain.  Appears uncomfortable and is holding abdomen. | | | |
| **Actors and roles in the room at case start** | **Patient** is in room at the start. Patient can be played by a learner or a standardized participant.  **Attending** physician (played by faculty or resident), enters room with team.  **Learners** initially are briefed together before starting the role play. All learners are given a description of the scenario, the backstory for the role of the Attending, and a description of the patient’s initial presentation.  **Attending**: Played by an instructor (faculty or resident).  **Action**: Leads team of learners into room. Informs team of need for speed on rounds, as the team has a surgical case scheduled to start in 15 minutes.  **Attending States**: “Okay we need to see this last patient and get to the OR. This is the patient that came in a few hours ago. I got sign-out from the on-call attendant. They worked the patient up for appendicitis, but it was negative, and the patient is good to be discharged. Did anyone pre-round on this patient?”  **Patient: Learner 1 or another instructor (attending or resident)** has the role of the patient. If it is Learner 1, they are briefed separately from the other learners. | | | |
| **HPI** | The patient is a 38 y.o. person in previously good health who presented to the Emergency Department early this morning with worsening nausea and abdominal pain. The pain began suddenly, was initially generalized, but settled in the right lower quadrant, and was rated as a 9/10. The patient came into the ED when vomiting developed, accompanied by their 17 yo son. The patient has limited English proficiency and requires an interpreter.  The patient was afebrile on presentation, and a tender abdomen, with no CVAT, rebound or guarding, but had an elevated WBC (12.6K). There were no other significant lab abnormalities. The overnight team was initially concerned for appendicitis and ordered a CT of the abdomen and pelvis; however this was negative for appendicitis, and the overnight team signed out to the day team that the patient was ready for discharge.  The team rounds on the patient prior to discharge and discovers their condition has deteriorated, with signs of acute abdomen, tachycardia, and hypotension. The decision is made to take the patient for urgent exploratory surgery for suspected appendicitis. The attending attempts to communicate with the patient without an interpreter. | | | |
| **Past Medical/Surgical History** | **Medications** | **Allergies** | **Family History** | Social History |
| No medical history  No surgical history  4 children | None | None | Parents in good health in home country in their 70s | Documented immigrant from non-English speaking country.  Married to undocumented immigrant. 4 children who are US citizens.  works full-time at a job at $15/hour, no health insurance. Supports parents with monthly checks. Speaks little English and prefers to use an interpreter for significant communication. |
| **Physical Examination** | | | | |
| **General** | Appears unwell and in pain, resting in bed, holding abdomen | | | |
| **HEENT** | Unremarkable to gross examination | | | |
| **Neck** | No stridor or jugular venous distention, neck supple. No thyromegaly or lymphadenopathy | | | |
| **Lungs** | Clear to auscultation bilaterally, no crackles or wheezes, no respiratory distress. | | | |
| **Cardiovascular** | Normal rate, irregular rhythm, no murmur | | | |
| **Abdomen** | Decreased bowel sounds, tender throughout, non-distended, + involuntary guarding, + rebound, no masses, no CVA tenderness | | | |
| **Skin** | Warm dry, no rashes or erythema | | | |
| **GU** | deferred | | | |

| **Instructor Notes - Changes and CASE Branch Points** | | |
| --- | --- | --- |
| **Intervention/Timeline**  Version 1 | **Change in Case/Trigger to Move to next frame** | **Learning Cues** |
| Simulation Start  Minutes 0-1  Attending asks learners to present the patient’s case,  Attending introduces self only to the patient, speaking in English.  Patient responds by indicating they do not speak English, states language they do speak | Learners must:   - Give the patient information from cards - Recognize attending is attempting to communicate without an interpreter. - Offer to call interpreter | Case goes to next time point |
| Minute 1-2  Attending dismisses offer to get interpreter, citing time constraint | Learners must:   - Recognize the Attending is attempting to communicate without an interpreter. - Offer to call interpreter |  |
| Branch point 1:  Attending asks if anyone on team speaks the patient’s language | Learners must:  Acknowledge own skills and limitations with language of patient (from role descriptions) | If participant(s) offer to act as interpreter, **follow Scenario 2** |
| Minutes 1-2  Attending begins taking interval history and examining patient in English.  Patient is cooperative but appears concerned and in pain, answers “yes” to all questions posed in English  Heart Rate: 112  Temp: 101.8 (38.8)  BP 94/58  RR 14  O2 sat: 99%  Patient acts out findings on Physical exam (rebound, guarding, pain in RLQ) Attending describes absence of bowel sounds on exam | Learners must:   - Suggest calling for interpreter - Use one TeamSTEPPS® communication tools (DESC or CUS) to communicate to attending (First Challenge of 2-Challenge Rule)   *The following are communication tools that may be used at any point, in either order*  *DESC Tools*  *-Describe the situation: the patient does not seem to understand us (give data, for example, they are answering yes to every question)*  *-Express concerns (I am worried the patient does not understand how sick they are)*  *-Suggest alternative (I can call an interpreter on my cell)*  *-Consequences/consensus (Calling an interpreter will save us time; one of us can stay to meet with the interpreter and the rest of the team can go to the OR; etc.)*  *C-U-S Words*  *-* I am *C*oncerned the patient does not understand us  *-* I am *U*ncomfortable about not calling an interpreter  *-*This is a patient *S*afety issue | If learners do not suggest interpreter, attending asks if learner will interpret, or there is a family member or hospital staff member (non-interpreter) available to interpret |
| Minutes 3-5  Attending responds by calling attention to patient’s vital signs and exam findings, begins discussing signs of sepsis. | Learners must:   - Suggest calling for interpreter - Use a second TeamSTEPPS® communication tools (DESC or CUS) to communicate to attending - (2nd Challenge of 2-Challenge Rule) | If learners do not use 2nd tool scenario continues  If learners do use 2nd tool **skip to time point Minute 7** |
| Minutes 5-7  attending informs patient in English of need for hospitalization and surgery for presumed ruptured appendix  Patient appears concerned but responds “okay, yes” and smiles | Learners must:   - Suggest calling for interpreter   State: This is a patient safety issue, I am concerned/not certain the patient understands they need surgery. | If learners do not use either TeamSTEPPS® tool, Attending tells team rounds are completed and instructs the senior resident role to add the procedure to the OR schedule and the intern to write pre-op orders. Scenario ends. |
| Minute 7  Attending acknowledges that patient does not understand information and agrees to call an interpreter. |  | Attending begins to acknowledge the patient’s pain, expresses empathy toward patient, thanks learner for calling for interpreter. |

| **Instructor Notes - Changes and CASE Branch Points** | | |
| --- | --- | --- |
| **Intervention/Timeline**  **Version 2** | **Change in Case/Trigger to Move to next frame** | **Learning Cues** |
| **Branch point 1**:  Attending asks if anyone on team speaks the patient’s language | Learners must:   - Begin “interpreting” for attending - Remind attending of need for formal interpreter - Acknowledge own skills and limitations with language of patient (one learner role has experience from living in country and considers themselves “fluent”, another learner role has family members who speak same language but does not have fluency) | If learners do not acknowledge limitations scenario continues |
| Minutes 1-2  Attending begins taking interval history and examining patient in English.  Patient is cooperative but appears concerned and in pain. Gives symptom details from role play card to learner who “interprets”  Heart Rate: 112  Temp: 101.8 (38.8)  BP 94/58  RR 14  O2 sat: 99%  Patient acts out findings on Physical exam (rebound, guarding, pain in RLQ) Attending describes absence of bowel sounds on exam  Minutes 3-5  Attending calls attention to patient’s vital signs and exam findings, begins discussing signs of sepsis. | Learners must:   - suggest calling for interpreter - use one TeamSTEPPS® communication tools (DESC or CUS) to communicate to attending (First Challenge of 2-Challenge Rule)   *The following are communication tools that may be used at any point, in either order*  *DESC Tools*  *-Describe the situation: the patient does not seem to understand us (give data, for example, they are answering yes to every question)*  *-Express concerns (I am worried the patient does not understand how sick they are)*  *-Suggest alternative (I can call an interpreter on my cell)*  *-Consequences/consensus (Calling an interpreter will save us time; one of us can stay to meet with the interpreter and the rest of the team can go to the OR; etc.)*  *C-U-S Words*  *- I am Concerned the patient does not understand us*  *- I am Uncomfortable about not calling an interpreter*  *-This is a patient Safety issue*  ***Return to version 1 timeline if TeamSTEPPS® communications tools are employed by any learner*** | If learners do not suggest interpreter or use TeamSTEPPS® communication skills, scenario continues |
| Minute 5-7  With learner acting as interpreter, Attending informs patient of need for hospitalization and surgery for presumed ruptured appendix.  Patient appears concerned, asks in their language, how much is this going to cost? Attending responds that the patient should not worry about that. The patient declines treatment and expresses intention of leaving. | Learners must:   - suggest calling for interpreter a second time - use 2nd TeamSTEPPS® communication tools (DESC or CUS) to communicate to attending (Second Challenge of 2-Challenge Rule)   ***Return to version 1 timeline if TeamSTEPPS® communications tools employed by any learner*** | If learners do not successfully use TeamSTEPPS®, attending states, this patient wants to leave against medical advice. Attending instructs intern to enter d/c orders and the rest of the team to head to the OR for the first case. |

**Ideal Scenario Flow**

Learners enter the room with the attending. They find a patient on a gurney, clearly in discomfort. The attending quickly introduces themself and briefly acknowledges the team. The attending informs the team that they need to see this patient quickly, as they are late to the operating room. The attending states the patient was signed out as ready for discharge by the night team and asks if anyone on the team has seen the patient this morning. The intern and medical student identified as having seen the patient give a short summary that the patient was tachycardic, febrile, and had an exam notable for rebound and guarding. The attending then begins to examine the patient and ask questions of the patient in English, acting oblivious to the patient’s pain and to the patient’s clear inability to communicate in English. One learner suggests they should call for an interpreter. The attending brushes this aside and says “Doesn’t anyone on the team speak this language?” The two team members who do speak the language volunteer that they do not feel comfortable interpreting and would prefer to call for an interpreter. The attending then notices the abnormal vital signs and performs a mock exam on the patient demonstrating rebound. The attending begins to ask the students questions regarding what they think the diagnosis is, the definition of sepsis, the definition of septic shock, and other valid teaching points, based on the clinical scenario. The attending then turns to the patient and asks the learners whose characters speak the language to interpret, to inform the patient that they are not able to be discharged at this time, and instead need exploratory surgery for a possible ruptured appendicitis. These learners again recommend obtaining an interpreter, using the TeamSTEPPS language and referring to this as a patient safety issue. The attending then acknowledges the lapse in professionalism and agrees to call the interpreter to communicate with the patient.

**Anticipated Management Mistakes**

1. Learner (s) agree to interpret. This is a common reaction and version 2 of the scenario describes the Attending character’s response. Learners who do agree to interpret should inform attending and team of their language limitations. When this happens, the scenario is allowed to continue until the learners eventually realize the attending has not communicated with the patient and the patient thinks they are going home. In our experience, no more than 2 to 3 minutes elapsed before a participant will realize the need to call for the interpreter regardless of the attending’s behavior.

2. Failure to act as a team. Learners who act together to intervene in attending’s conversation and examination of the patient are more effective at communicating within the hierarchical team.

3. Failure to ask clarifying questions/unfamiliarity with roles on medical teams. Pre-clerkship students may not be familiar with the different roles of a senior resident, attending, intern, student on a medical team. We anticipate learners will have limited knowledge of the roles and responsibilities of a team and will need to ask about details of the roles. Learners commonly try to complete the exercise without clarifying the expectations of the roles. This learner response to the simulation contributes to the poor communication within the session. Facilitators can illuminate this in the debrief. We modified the pre-scenario briefing to include a brief description of the roles of the different players, in addition we instruct learners to share their character role descriptions in the briefing to identify and address any lapses in knowledge.

4. Failure to obtain patient social history. Although not vital to completing the exercise, teams that obtain the patient’s social history have a better understanding of the patient’s reactions to treatment recommendations.

**Flowchart- see Appendix D**
