# Supplementary material for: “Should I Say Something?”: A Simulation Curriculum on Addressing Lapses in Professionalism to Improve Patient Safety
Source: MedEdPORTAL. 2023 Dec 12;19:11359. doi: 10.15766/mep_2374-8265.11359 (PMC10713868; doi:10.15766/mep_2374-8265.11359)
Supplement: Supplementary file 1 — Case Summary.docxNarrated Preclass Presentation.m4vCharacter Role Cards.docxFlowchart for Simulation Role-Play.pdfBrief and Debrief Guide.docxCritical Actions Checklist.docxSISS Pre- and Postsurveys.docx [file mep_2374-8265.11359-s001.zip › E. Brief and Debrief Guide.docx]

**Appendix E: Briefing and Debriefing Guide**

**Briefing information for facilitators:**

Students have worked together in case-based, collaborative learning groups during the first year of medical school. This simulation is one of three stations on Skills Day, which students attend in their Academic Society groupings. Students may have differing levels of interpersonal relationships based on these experiences, ranging from acquaintance to close friendships, which may affect their participation in the simulation role play.

Students may have a limited knowledge of clinical workflow and responsibilities of members of a patient care team. A quick orientation as to how inpatient teams function is helpful (for example, pre-rounding, job descriptions of interns vs. residents, bedside presentations, etc.).

Although this simulation uses a Surgical team, it may be modified to a Medicine team if you are more comfortable with that structure.

Inform students that the exercise is formative, and they will receive no grade or feedback other than the debriefing.

In the following Script, actions are underlined.

**Script:**

Read to participants:

Welcome to simulation day. In this exercise we will have the opportunity to practice communication in healthcare teams using skills from TeamSTEPPS®. Students often are not sure when or how to speak up about perceived errors or unprofessional behavior and may feel unqualified to identify these issues. This scenario involves a commonly witnessed lapse in professionalism, which can have direct impact on patient care, and which students are completely capable of recognizing: not using an interpreter for a patient with limited English proficiency.

Teams need to check in on hospitalized patients every morning, with a brief interval history, a focused physical exam, and a review of any new data. Rounds may also include a social worker, pharmacist, or nursing staff. Usually each trainee will “pre-round” on their assigned patients and later present the patient’s case to the entire team. The team discusses the patient’s care and plan for the day. As a student, you will be expected to see one or two patients and present them on rounds, including your interpretation of the clinical findings, a problem list, and a plan of care.

In this case, the health care team consists of an Attending, a senior resident, an intern, a sub-intern, a clerkship student, and the Patient. The team is in a hurry to see all the patients before the day’s planned activities, and the Attending cuts some professional corners, placing patient safety in jeopardy. The goal of the exercise is to successfully communicate to the Attending that the behavior is not meeting professional standards and potentially endangering the patient, getting the Attending to change this behavior. You can work together to achieve this goal.

Each of you will have a role randomly assigned, which will come with detailed instructions and a backstory. One of you will receive the role of Patient. Dr. X will be the Attending. The simulation will start when the team comes into the patient’s room and will conclude when the Attending accepts the team’s recommendations to call for an interpreter.

Allow participants to each pick one of the laminated Character Cards.

Read to participants: Everyone who is not the Patient please step into the other room.

*Separate briefing for Patient Role:*

*Allow student to read character card. Confirm student is comfortable with this role.*

*Dr. X will lead the team back into this room to make rounds. You can sit or lie down on the stretcher.*

*Dr. X will interact with you and will mime an abdominal exam. Dr. X will lead the team in a discussion of your care and will explicitly ignore you during the discussion. Dr. X will tell you the plans but will not confirm your comprehension. You can ask for an interpreter and interact with the other team members.*

*Separate briefing for Team Roles:*

*Dr. X will lead the team back into the room to make rounds. Please respond to Dr. X’s questions as you think your character might. Dr. X will interact with the Patient and will mime an abdominal exam. Dr. X will lead the team in a discussion of the patient. You can work together or independently to communicate with Dr. X, with the goal of calling for an interpreter for the patient care episode.*

**Recommended debriefing strategy:**

· Identify the end of the role play definitively, for example stating, “that’s the end of the role play”.

· Begin with open-ended questions to assess the learners’ emotional state and initial reactions to the exercise.

- Ask, “How do you think that went?” to each participant. We recommend starting with the student who played the Patient role, followed by the student who was the most participatory during the exercise. Guide students to limit their comments, so all participants get a chance to speak. It is helpful to take notes of points made by participants to ensure all concerns are addressed.
- Ensure the student who played the Patient has adequate time to speak. This student often has new insights into the patient experience to share.

· The Advocacy-Inquiry technique incorporates facilitator observations to prompt reflection. The Facilitator or Observer can note an observed behavior or interaction, and ask the participant to elaborate on the experience:

- “I noticed the students playing the roles of the Intern and Resident did not react when the student playing the role of New Clerkship Student suggested calling an interpreter. Can you tell me what your thoughts were then?”
- “The Patient seemed to be in a great deal of pain. Can you explain how that made you feel? What did you think of the Attending’s lack of response to the Patient?”

Allow 3-4 minutes for responses and discussion.

· Ask if participants have any reflections they would like to share. The character backstories may resonate with students and prompt sharing of their own or their families’ experiences. Point out the simulation is intended to give students tools to communicate and advocate effectively in a hierarchical environment.

· If a theme resonates with the group (for example, feeling dismissed by the Attending’s behavior, or the Patient’s experience of being excluded from the discussion), ask for suggestions as to how to address the concerns in the moment.

· Students are often concerned speaking up will affect their grade or assessments on clerkships. Remind students: At our institution, there is NO penalty for speaking up, and in fact student advocacy is expected. Reassure students that only the Clerkship Director can assign a grade for a clerkship, and that Clerkship Directors and administrators are available if students have concerns about retaliation or other consequences of speaking up on professionalism lapses.

**Correct any misconceptions/errors. The following are common areas of confusion:**

The debrief facilitators should correct any errors and provide best-practice recommendations for learners. The following table highlights common errors and misconceptions learners may express during debrief, with references for facilitators.

| Barrier to Communication | Solutions and Recommendations | References |
| --- | --- | --- |
| **Inconvenience/Time**  *“If it’s two in the morning, can we find an interpreter for this patient’s language?”* | 1. Most hospitals contract with interpreter services which can locate a certified interpreter for phone or video call interpretation 2. If possible contact the service as soon as the need for an interpreter is identified, and give estimate for time required and future needs | Garcia ME, Mutha S, Napoles AM, Malevanchik L, Williams M, Karliner LS. "Long Overdue": Nurse and Resident Physician Perspectives on Implementation of Dual-Handset Interpreter Phones in the Inpatient Setting. Health Equity. 2023 Feb 16;7(1):100-108. doi: 10.1089/heq.2022.0023. PMID: 36876231; PMCID: PMC9982138.  Tuot DS, Lopez M, Miller C, Karliner LS. Impact of an easy-access telephonic interpreter program in the acute care setting: an evaluation of a quality improvement intervention. Jt Comm J Qual Patient Saf. 2012 Feb;38(2):81-8. doi: 10.1016/s1553-7250(12)38011-2. PMID: 22372255. |
| **Accommodations for Disability**  *“Do patients with deafness who lip read need interpreters?”* | In the US, American Sign Language (ASL) interpreters are required to be available for patients with disability due to hearing loss or deafness under the Americans with Disabilities Act (ADA). | Ali S. Providing interpreters for patients with hearing disabilities: ADA requirements. Innov Clin Neurosci. 2012 Sep;9(9):30-3. PMID: 23074701; PMCID: PMC3472899. |
| **Social Network and Family**  *“Can we ask a patient’s child who is a teen-ager to translate if they speak English well?”*  *“Can we ask a patient’s sister-in-law who the patient is close to, if they speak English well?”* | Family members and especially children should never be used as interpreters.  Medical accuracy, privacy and safety concerns exist when family members are asked to interpret. | Free C., Green J., Bhavnani V., Newman A.: Bilingual young people's experiences of interpreting in primary care: a qualitative study. Br J Gen Pract 2003; 53: pp. 530-535. |
| **Use of Technology**  *“Can I use this translation app on my phone?”* | Online automated translation services are generally not recommended for both privacy and accuracy concerns. | Chang, Dwayne, and Melissa Maluda. "Uses of Mobile Phone Language Translation Applications in Surgery." Journal of Urological Surgery 8.4 (2021): 238-242. |
| **Communication during Emergencies**  *“What happens in an emergency if we do not have time to call an interpreter”* | In cases of true life-threatening emergencies (cardiac or respiratory arrest, fetal compromise, massive hemorrhage), healthcare providers communicate with patients/family as best as possible.  Best practice is to acknowledge the emergency to the patient/family with reassurances that as soon as an interpreter is available a member of the team will speak with the family/patient. | Lundin C, Hadziabdic E, Hjelm K. Language interpretation conditions and boundaries in multilingual and multicultural emergency healthcare. BMC Int Health Hum Rights. 2018 Jun 5;18(1):23. doi: 10.1186/s12914-018-0157-3. PMID: 29866163; PMCID: PMC5987383. |
| **Humanism concerns**  *“I don’t speak this patient’s language, and they seem to be suffering- is it okay to try to communicate anyway?”* | Reassure students it is always appropriate to offer comfort to patients/families. |  |
| **Learners or Staff as Translators**  *“What if the med student speaks this patient’s language, maybe they can translate for the team.”* | If the student does have language concordance, communicating in the patient’s language is acceptable, however using a professional interpreter is preferred, as students may not know medical terms or have complete fluency. | Yang C.-F., Gray B.: Bilingual medical students as interpreters: what are the benefits and risks? N Z Med J 2008; 121: pp. 15-28. |

**Questions to prompt reflection:**

What are some systems-based improvements that might promote patient safety regarding interpreter services?

If a patient-safety issue is witnessed and a student does not feel comfortable addressing in the moment, what are other steps that can be taken (for example, anonymous or confidential reporting, discussion with a friend, mentor, or advisor, discussing with nursing staff or other health professional).

**End with a request for feedback on the exercise and remind students to complete the post-simulation assessment.**
