# Supplementary material for: “Should I Say Something?”: A Simulation Curriculum on Addressing Lapses in Professionalism to Improve Patient Safety
Source: MedEdPORTAL. 2023 Dec 12;19:11359. doi: 10.15766/mep_2374-8265.11359 (PMC10713868; doi:10.15766/mep_2374-8265.11359)
Supplement: Supplementary file 1 — Case Summary.docxNarrated Preclass Presentation.m4vCharacter Role Cards.docxFlowchart for Simulation Role-Play.pdfBrief and Debrief Guide.docxCritical Actions Checklist.docxSISS Pre- and Postsurveys.docx [file mep_2374-8265.11359-s001.zip › F. Critical Actions Checklist.docx]

**Appendix F: Critical Actions Checklist**

| **Critical Actions Checklist** | **Completed correctly? Y/N** |
| --- | --- |
| **1. Recognizes language discordance between Attending and Patient** |  |
| **2. States own character’s language abilities (found in Character description)**  **Circle action of participant** |  |
| None |  |
| Informal (family speaks same language, character speaks/understands some) |  |
| Self-identified ‘fluency’ (lived and worked in language-concordant country) |  |
| **3. Offers to call medical interpreter.** |  |
| **4. Uses TeamSTEPPS® communication tools (check all that apply)** |  |
| [ ] C-U-S |  |
| [ ] DESC-ribe |  |
| [ ] Two Challenge Rule |  |
| **5. Actions to avoid:** | **Performed? Y/N** |
| [ ] Offer to interpret without adequate training |  |
| [ ] Use online automated translation tool |  |
| [ ] Agree to use Patient’s child as interpreter |  |
