# Supplementary material for: “Should I Say Something?”: A Simulation Curriculum on Addressing Lapses in Professionalism to Improve Patient Safety
Source: MedEdPORTAL. 2023 Dec 12;19:11359. doi: 10.15766/mep_2374-8265.11359 (PMC10713868; doi:10.15766/mep_2374-8265.11359)
Supplement: Supplementary file 1 — Case Summary.docxNarrated Preclass Presentation.m4vCharacter Role Cards.docxFlowchart for Simulation Role-Play.pdfBrief and Debrief Guide.docxCritical Actions Checklist.docxSISS Pre- and Postsurveys.docx [file mep_2374-8265.11359-s001.zip › _Educational Summary Report_11359.pdf]

MedEdPORTAL® | The AAMC Journal of Teaching and Learning Resources

Original Publication

Open Access

"Should I Say Something?": A Simulation Curriculum on Addressing Lapses in Professionalism to Improve Patient Safety

Lydia A. Flier, MD, Jeremy B. Richards, MD, MA, Michele R. Hacker, ScD, Alexandra Hovaguimian, MD, Anita Vanka, MD, Amy Sullivan, EdD, Celeste S. Royce, MD*

*Corresponding author: croyce@bidmc.harvard.edu

Abstract

Introduction: Medical students may witness lapses in professionalism but lack tools to effectively address such episodes. Current professionalism curricula lack opportunities to practice communication skills in addressing professionalism lapses. **Methods:** We designed a simulation curriculum to introduce professionalism expectations, provide communication tools using elements of the Agency for Healthcare Research and Quality TeamSTEPPS program, and address observed professionalism lapses involving patient safety in hierarchical patient care teams. Students were surveyed on knowledge, skills, and attitude regarding professionalism before, immediately after, and 6 months after participation. **Results:** Of 253 students, 70 (28%) completed baseline and immediate postsurveys, and 39 (15%) completed all surveys. In immediate postsurveys, knowledge of communication tools (82% to 94%, $p = .003$) and empowerment to address residents (19% to 44%, $p = .001$) and attendings (15% to 39%, $p < .001$) increased. At 6 months, 96% of students reported witnessing a professionalism lapse. **Discussion:** The curriculum was successful in reported gains in knowledge of communication tools and empowerment to address professionalism lapses, but few students reported using the techniques to address witnessed lapses in real life.

Keywords
Interpreters, Communication Skills, Flipped Classroom, Professionalism, Quality Improvement/Patient Safety, Role-Play/Dramatization, Simulation

Educational Objectives

By the end of this activity, learners will be able to:

1. List five key components of professional behavior related to patient safety.
2. Define the three TeamSTEPPS communication tools (CUS, two-challenge rule, and DESC-ribe).
3. Describe one challenge and one benefit to speaking up about lapses in professional behavior related to patient safety.
4. Employ TeamSTEPPS communication tools to communicate within a medical team.

Citation:
Flier LA, Richards JB, Hacker MR, et al. "Should I say something?": a simulation curriculum on addressing lapses in professionalism to improve patient safety. *MedEdPORTAL*. 2023;19:11359.
https://doi.org/10.15766/mep_2374-8265.11359

Introduction

Lapses in professionalism affect patient safety, adverse outcomes, and medical errors and contribute to adverse learning environments.[1,2] Learners report witnessing unprofessional behaviors more often than traditional patient safety concerns (such as medication errors) but recount speaking up about professionalism issues less frequently, identifying fear of conflict as a barrier.[3] Learners also report lacking the skills and knowledge to address their concerns about professionalism lapses.[4] Learners are less likely to speak up when a professionalism lapse is committed by an attending physician and more likely to speak up in an environment perceived as being supportive, safe, and one in which teamwork and patient safety are valued.[3,5] Instruction in professionalism skills and behaviors before students' clinical responsibilities start has long-term benefits, as it is associated with decreased unprofessional behavior in later careers.[6] However, opportunities to learn and practice strategies to address conflict and unprofessional behavior are limited in the standard medical curriculum. To date, there is no standardized approach or recommended timing in the medical school curriculum for teaching professionalism or

Copyright © 2023 Flier et al. This is an open-access publication distributed under the terms of the Creative Commons Attribution license.

approaches to address observed lapses in professionalism, and curricula reported in the literature are aimed at remediation of unprofessional behaviors, not prevention.[7,8]

Students in the preclerkship phase of medical education have limited exposure to medical teams and may be more likely to identify lapses in professionalism, as they have not acquired the desensitization to breaches in professionalism that are part of the hidden curriculum.[9-12] Once in the clinical learning environment, learners occupy a vulnerable place in the hierarchy of health care teams. While they are expected to behave professionally and engage in safe patient care, learners feel they are continuously being evaluated and may be reluctant to speak up. Unfamiliarity with health care team member roles, frequent changes in team members, and dependence on supervising personnel for future career goals may contribute to a sense of risk and perceived cost for speaking up.[13] In addition, medical students may not be familiar with recommended communication techniques for hierarchical teams, such as crew resource management, as they are not often included in team training exercises. In this context, we describe a simulation curriculum that addresses this gap, in which students learn and practice skills necessary to communicate within a health care team to address observed lapses in professionalism within a framework of patient safety principles. By using the TeamSTEPPS tools, we sought to provide learners with effective communication strategies they could practice using to confront unprofessional behavior and minimize such behavior's impact on patient safety.

In developing this curriculum, we chose to appeal to students' sense of altruism, based on the theory of self-determination.[14] This framework centers on intrinsic motivation, wherein learners engage in an activity out of genuine interest. It is associated with deeper learning, better performance, and improved well-being. Altruism is highly valued as a core component of intrinsic motivation by many medical students and physicians.[14,15] In designing the simulation scenario, we consulted the literature in which professionalism is taught by defining expectations of observable behaviors, which then provides the basis for assessment, feedback, and determination of progress toward desired outcomes.[16,17] Hundert and colleagues suggest role-play as an effective tool for promoting ethical development in physicians.[18] Following these principles, we sought to establish observable actions consistent with professional behavior norms for both students and faculty and used simulation-based role-play as the pedagogical technique. We decided on team-based learning to leverage the concept of communities of practice derived from situated cognition theory.[19] This model emphasizes the social aspect of learning, in which context and community influence learning and thinking.

The clinical clerkship year is recognized as a time when medical students' empathy and idealism regarding professionalism wane.[6,20,21] This course was developed for implementation immediately prior to the clerkship year to influence students' perceptions and behaviors prior to witnessing unprofessional behaviors. Based on its efficacy for teaching professionalism,[18,22,23] simulation role-play was chosen as an effective pedagogic approach for learners at this stage.[23,24] Simulation also provides the opportunity for guided reflection on behavior and performance through structured debriefing, which promotes reflective learning and practice, integral components of professional growth and behavior change.[24-26]

To focus on the patient safety implications of lapses in professionalism, our simulation content replicated a commonly observed occurrence: a physician not calling for an interpreter when evaluating a patient with limited English proficiency (LEP). Patients with LEP have worse outcomes, lower patient satisfaction, and decreased quality of care when interpreters are not incorporated into the care team.[27] Use of interpreters is a recognized standard in patient care and can serve as an expected and observable professional behavior. We designed a case in which a medical student could be reasonably expected to intervene to address a professionalism lapse within a hierarchical team. We created a case with a clear correct answer (obtaining an interpreter for a patient with LEP) to minimize any effect of asymmetrical knowledge or medical nuance that might inhibit speaking up.

Using patient safety and prevention of medical error as the centerpiece, we designed and implemented a novel simulation-based curriculum to introduce concepts of professionalism to preclinical medical students. Current published curricula for teaching professionalism in undergraduate medical education include self-study modules, facilitated discussion, didactic lectures, and video vignettes.[28-30] To our knowledge, there are no published simulation curricula for teaching professionalism for medical students, and our curriculum addresses this gap.

Methods

Development

Harvard Medical School instituted the Pathways curriculum in 2015-2016, moving clerkships to the second year of the medical school curriculum, with an entirely flipped classroom, case-based, small-group-discussion format.[31] The process of relocating the

Copyright © 2023 Flier et al. This is an open-access publication distributed under the terms of the Creative Commons Attribution license.

clerkships to the second year of medical school was staged to create a 6-month overlap of clerkship students from the prior curriculum (third-year students) and the new curriculum (second-year students). A needs assessment after the 6-month overlap asked clerkship directors to identify competency domains where students entering clinical clerkships after this shortened preclerkship curricular phase might need additional preparation. We identified recognition of professional behavior as an area requiring better preparation. Based on these data, we defined three goals for this simulation curriculum:

1. Provide students with knowledge of professional behavior expectations for themselves and health care team members.
2. Give students tools for addressing observed lapses in professionalism by a team member in real time.
3. Provide an opportunity to practice using those tools in a safe environment.

For complete case details, please refer to the simulation case summary (Appendix A). We developed the simulation with a flipped classroom model, including a narrated 25-minute slide set (Appendix B). The preclass material was available to the students up to 4 weeks prior to participation in the simulation. The simulation session, including orientation, role-play, and debriefing, lasted 45 minutes. The simulation was one of three stations in a simulation skills day offered weekly as part of a 4-week course in the month immediately prior to starting clinical clerkships. The skills day included a 25-minute orientation at the start of the day; students then completed the simulation and debrief in groups of four to five. We offered the curriculum in 2018 and 2019 and suspended the course in 2020 due to COVID-19 constraints on in-person gathering and staffing shortages.

The prerecorded lecture and slide set consisted of four sections. In the first section, we reviewed definitions of professionalism and highlighted expected professional behaviors for clerkship students. Students were prompted to consider potential differences between their personal definitions of professionalism and the aspects of professionalism that residents and faculty might assess. The second section described qualities of successful teams, including shared goals, mutual support, and using the SBAR (situation, background, assessment, and recommendations) handoff tool as a common mnemonic for effective interprofessional communication.[32] The third section introduced TeamSTEPPS, focusing on three techniques for conflict resolution: CUS words (I am concerned, I am uncomfortable, this is a patient safety issue), the DESC-ribe model (describe, express, suggest, consequences), and the two-challenge rule.[33] We chose to emphasize these techniques for their utility in catalyzing effective communication within hierarchical teams that students might encounter in clerkship settings. The techniques provided junior team members with a patient safety–centered approach to challenging senior team members. This approach focused on behavior, rather than personal characteristics, which could be more effective in achieving desired behavioral change. The fourth section of the preclass material consisted of video vignettes demonstrating these techniques as possible responses to witnessed lapses in professionalism.

Equipment/Environment

The simulation took place in our institution's simulation center, utilizing a simulated single-bed hospital room with capacity for electronic vital signs display monitors and a one-way observation window. The role-play lasted approximately 7 minutes, followed by a 15- to 20-minute guided debriefing. Other equipment included a white coat and stethoscope for the attending physician. Each participant was randomly assigned a role as a member of the team and received a laminated card describing their role and their character's backstory (Appendix C).

Personnel

The personnel required for this simulation included an individual familiar with the function and roles of health care teams to play the role of attending as a standardized participant. Using an actual physician proved beneficial due to the need to understand the way teams make rounds and the role of a supervising attending. A second person served as timekeeper and observer. In each session, the attending role was played by one of the authors. A member of the simulation center staff (one of two registered nurses) or the course administrator served as the observer. The observer interrupted the session with a knock on the door if the allotted time for a session had elapsed. The observer assisted in the debrief facilitation, as this individual monitored the team for nonverbal communications the attending may not have noticed. The observer remained behind the observation window during the simulation.

Implementation

For each skills day, participating faculty and simulation staff met for 5-10 minutes prior to the session to review the simulation, including timing, debrief checklist, and number of students participating. On the day of the session, one of the authors gave a brief, 20-minute review of the precourse material and a description of the role-play scenario as part of the skills day

Copyright © 2023 Flier et al. This is an open-access publication distributed under the terms of the Creative Commons Attribution license.

orientation. Students then participated in the simulation in groups of four to five, also taking part in other unrelated skills stations running in parallel.

At the time of the simulation, students received laminated role-assignment cards to read immediately prior to participation (Appendix C). A flowchart describing the simulation can be found in Appendix D. The roles included a new clerkship student, a subintern, an intern, a senior resident, and a patient. For groups of only four learners, we omitted the subintern role. The student playing the patient was instructed to wait in the room and read their role card. The faculty member escorted the remaining students to another room, instructed them to read their roles, and answered any questions. The student playing the role of the patient was briefed separately by the faculty member or observer. This student was instructed to act as a person who did not speak or understand English with enough proficiency to follow the group discussion. Importantly, this role did not require the student to speak or understand another language, merely to act as though they did not speak or understand English. The student was instructed to act as a reasonable person might, including trying to communicate with team members who might speak or understand their own language. All students were instructed that some roles in the scenario understood or spoke the patient's language and to interact accordingly. Faculty instructed learners to act as their character might during the simulation, and learners had access to their character cards for reference throughout. The faculty member playing the role of the attending reminded all students that the attending would display a lapse in professionalism and that student participants would have the opportunity to intervene.

The role-play began with the attending leading the team back into the room to round on the patient. The attending interacted with the patient using unidirectional communication, not recognizing the patient as LEP until learners intervened. The attending then used inappropriate means of communication (increasing speaking volume, miming, asking if a family member were available to translate, or using translation phone apps) and encouraged the participants to do so as well. The attending continued to interview and attempted to examine the patient until one or more learners used the complete set of communication tools or until the two-challenge rule was met. At that time, the attending agreed to call an interpreter before continuing with the patient encounter, and the scenario ended with the observer entering the room. The group moved to a debrief room where the faculty member and observer led the debrief. The debrief lasted 15-20 minutes and included learners' reflections and time for clarifying questions. The briefing and debriefing guides are in Appendix E.

Debriefing

The participating faculty member and observer conducted a short structured formal group debrief with all learners immediately following the simulation (Appendix E). The goals of the debrief were to ensure participant psychological safety, highlight the communication techniques used, and discuss successes and opportunities for improvement in team communication. First, we established learner well-being after the exercise. Second, we asked the participant playing the role of patient to describe their experience, followed by the other team members. A third component prompted self-reflection with the question "How do you think that went?" After a group reflection, the faculty member or observer reviewed when in the scenario the critical actions had occurred and how the team had communicated to the attending. Lastly, the participants had the opportunity to provide verbal feedback on the efficacy of the session.

Assessment

Learners completed the simulation session as a team, and thus, the critical actions checklist we developed (Appendix F) was limited to actions of the team. We developed a survey (Appendix G) to determine learners' acquisition and retention of knowledge, skills, and behaviors covering definitions of professional behavior and communication techniques (knowledge), use of communication techniques (skill), and whether they had employed any of those techniques (behavior). We conducted a literature search for similar instruments but did not identify one consistent with our specific objectives. We piloted the instrument among four postclerkship student volunteers who were not in the final respondent population and conducted cognitive interviews with one volunteer faculty member, revising the instrument after each round of input. Surveys used 5-point Likert scales and included free-text boxes for students to report personal experiences with observed lapses in professionalism. We administered baseline surveys 30 minutes before the simulation. Postsession surveys were administered immediately and at 6 months after the session. Surveys were distributed electronically and were voluntary and deidentified. To link baseline and later surveys, respondents were asked to choose a personal identifier including the last four digits of their home phone number. For analysis of the data, the Likert-scale options of strongly agree and agree were collapsed, and proportions were compared using the McNemar test. This project was initially approved as a pilot curriculum by the Harvard Medical School Office of Educational Quality Improvement and later was deemed an educational quality improvement project.

Copyright © 2023 Flier et al. This is an open-access publication distributed under the terms of the Creative Commons Attribution license.

Results

In 2018 and 2019, 270 students were scheduled to attend the simulation as part of a transition to the clerkship year preparatory course. Of the 253 (94%) who attended, 179 started the baseline survey (71%), and 72 finished it (28%). Seventy students completed the immediate postsurvey (28%), and 39 completed a 6-month postsurvey (15%). Demographic characteristics are listed in Table 1. Students who completed the baseline survey were similar to those who completed the immediate and 6-month postsession surveys with regard to age, gender, and self-reported race/ethnicity.

Between baseline and immediate postsurveys, there was a significant increase in the percentage of students who reported knowing how to communicate about patient safety issues (82% to 94%, $p = .003$; Table 2). There also was a significant increase in the percentage who felt empowered to speak up to residents (19% to 44%, $p = .001$) and attendings (15% to 39%, $p < .001$). Moreover, there was a significant increase in the percentage who agreed they were expected to speak up when they observed a lapse in professionalism (67% to 80%, $p = .02$). The percentage who agreed they were expected to speak up when there was a patient safety issue did not increase significantly, though this was in the setting of a high percentage of this expectation at baseline (94% to 97%, $p = .32$). None of these gains persisted at the 6-month survey (Table 2).

Student satisfaction with the curriculum was high. One hundred thirty-five students (53%) completed course evaluations, of whom 100 (74%) rated the curriculum as excellent and 25 (18%) as good. Of 103 narrative comments for the entire 5-week course (62 in 2018, 41 in 2019), 39 mentioned this session (32 in 2018, seven in 2019), and all were positive comments. The prework video had 473 viewings with 311 unique users, of whom 203 viewed once, 79 viewed twice, and 16 viewed three times.

Six months after the course, a majority of respondents reported they had learned either a moderate amount or a lot about the definition of professionalism (59%) and about how professionalism is assessed in clerkships (61%) from the simulation (Table 3). Overall, 70% of respondents reported satisfaction with the amount of training, 23% reported it was not enough, and 8% reported it was too much training.

In the 6-month postsurvey, 37 of 39 respondents (96%) reported witnessing a lapse of professionalism in their clerkships. Of these, 14% reported speaking up in response, using any of the techniques taught.

Table 1. Respondent Characteristics

Characteristic	No. (%)		
	Baseline (*N* = 179)	Immediate Post (*N* = 72)	6-Month Post (*N* = 39)
Age ≤25 years	149 (83)	58 (81)	30 (77)
Gender[a]			
Female	89 (50)	34 (47)	20 (51)
Male	86 (48)	37 (51)	19 (49)
Prefer not to answer/missing	4 (2)	1 (1)	0 (0)
Race/ethnicity			
African American	15 (8)	4 (6)	5 (13)
Asian	51 (28)	21 (29)	13 (33)
Hispanic or Latino	10 (6)	7 (10)	1 (3)
White	76 (42)	30 (42)	17 (44)
≥1 race/ethnicity or other	20 (11)	9 (13)	3 (8)
No answer	7 (4)	1 (1)	0 (0)

[a]Responses were limited to binary or prefer not to answer.

Discussion

We developed a simulation curriculum incorporating elements of role-play and flipped classroom to teach concepts of professionalism and communication tools for preclerkship students and found improvement in self-reported knowledge, empowerment, and understanding of expectations around speaking up about professionalism lapses. Not all improvements reached statistical significance, however. These gains did not persist at a 6-month follow-up. Our curriculum was well received, with course evaluation data indicating that over 90% of participants rated the exercise as an excellent or good contribution to their learning, while narrative comments were uniformly positive. Course evaluation data may be reflective of a wider sample of the participants than our survey responses, as the response rate was higher.

Our survey responses highlight the commonality of professionalism lapses witnessed by students, with 96% of respondents at 6 months reporting a witnessed lapse, consistent with published surveys.[34] Our finding that only 14% reported using the strategies taught in the simulation is also consistent with the literature. In their study of student responses to professionalism breaches, Mak-van der Vossen and colleagues reported low numbers for the suggested response strategies of ignore, challenge individual, discuss with peers, or report.[4] They that noted students chose to ignore lapses when they did not know how to respond and that, after avoiding a lapse, students without exception reported discussing the observed lapse with peers. The authors suggested that although students choose to ignore a lapse in the moment, they may be motivated to other action, such as seeking change in policies, commenting that "these student leaders are likely to be the future change agents the medical profession needs."[4] In our 6-month survey, 82% of

Copyright © 2023 Flier et al. This is an open-access publication distributed under the terms of the Creative Commons Attribution license.

Table 2. Responses of Strongly Agree or Agree on the Presession and Immediate Postsession Surveys

Item	No. (%) Baseline ($N = 72$)	Immediate Postsession ($N = 70$)	p
I know how to communicate about patient safety issues	59 (82)	68 (94)	.003
I feel empowered to speak up when I witness a lapse in professionalisms:			
Among residents[a]	14 (19)	30 (44)	.001
Among attendings	11 (15)	26 (39)	<.001
It is expected I will speak up if I witness:			
A lapse in professionalism	48 (67)	56 (80)	.02
A patient safety issue	67 (94)	68 (97)	.32

[a]Percentages on this item do not add up to 100% due to incomplete responses.

respondents agreed they had learned new strategies to address lapses in professionalism. This suggests longer-term retention of these skills that students can employ in innovative, systems-level approaches to improving professionalism, as suggested by Lucey and Souba.[35]

Our follow-up finding that most respondents chose not to address a professionalism lapse may be due to several causes. Perhaps a one-time intervention was insufficient to motivate action and additional interventions are needed to improve learners' confidence in and ability to address professionalism lapses. Another possibility is that the hierarchies of medicine or concerns about grades continue to inhibit student responses. Professional identity formation and concepts of professionalism begin prior to matriculation and evolve during medical school. Elements of the hidden curriculum include unspoken and untaught expectations regarding professional behavior.[18,33,36] These influences may have affected students' willingness to use the communication skills taught without affecting their acquisition of knowledge of the techniques. This is supported by the finding that students did report increased empowerment at the immediate postsurvey. Alternatively, students' low hierarchical status on clinical teams and their unique vulnerability as learners seeking positive evaluations may have affected their behavior.[37] More completely understanding the reason for this finding represents an opportunity for future studies.

The role-play included two unique roles: the patient and the attending. During our first iteration, the role of the patient was played by simulation center staff or a volunteer faculty member. This role requires the participant to pretend to have a limited understanding of English and all participants to engage in a suspension of disbelief. Students suggested the role could be played by a learner to help learners understand the patient experience. We found learners in this role routinely reported similar experiences of feeling excluded from the conversation. Sharing this experience with their peers anecdotally elicited empathy for patients with LEP and others who have difficulty communicating (e.g., individuals with deafness, developmental delay, or dementia). All learners shared their experiences in the debrief, allowing the entire group to benefit from the lessons learned by each participant.

We elected to use physicians for the attending role as it required a detailed understanding of how in-patient rounds occur and of the roles on the team. Concerns might be raised about having

Table 3. Self-Described Learning—6-Month Postsession Survey ($N = 39$)

Item	No. (%) Little/No Learning	Moderate Learning	Learned a Lot
Definition of professionalism	16 (41)	17 (44)	6 (15)
Assessment of professionalism in clerkships	16 (41)	20 (51)	4 (10)
Role of professionalism in patient safety	7 (18)	22 (56)	10 (26)
Methods of addressing patient safety concerns	7(18)	25 (64)	7 (18)
	**Too Much**	**The Right Amount**	**Too Little**
The amount of time spent on this training was:	3 (8)	27 (70)	9 (23)
	**Yes**	**No**	
Since starting clerkships, I have witnessed a lapse in professionalism	37 (96)	2 (5)	
Since starting clerkships, I have spoken up about a lapse in professionalism ($N = 37$)	5 (14)	32 (86)	

Copyright © 2023 Flier et al. This is an open-access publication distributed under the terms of the Creative Commons Attribution license.

a member of the faculty participate in the role-play, in that learners could feel intimidated by working with a supervising physician and faculty member or that it may be disingenuous for the instructor to participate in the role-play as if unaware of the professionalism lapse acted out. Any role-play simulation requires participants to suspend disbelief. We believe using nonevaluative physicians contributes to the realism of the simulation; learners can practice skills in a realistic but safe setting with a faculty member giving feedback and suggestions for improvement. Additionally, the commonality of the professionalism lapse used in this simulation means most physicians can easily act out the role as they have most likely witnessed this lapse themselves. We do not believe either aspect adversely affected the learner experience or our results, and no participant comments indicated discomfort with faculty participation. The simulation can employ actors or nonphysicians as standardized participants for these roles if desired.

The group role-play exercise may have influenced the behavior of simulation participants. At our institution, preclerkship students learn almost exclusively in team settings, termed case-based collaborative learning.[31] Team-based learning is a widely accepted pedagogical approach, and students are unlikely to feel inhibited in this setting.[38] Additionally, the simulation reflects skills students need to work on interprofessional health care teams, fulfilling the Liaison Committee on Medical Education's stipulation for preparation to function on interprofessional teams.[39] Anecdotally, students in the simulation frequently worked collaboratively to achieve the goal of calling the interpreter.

Our sample size of respondents was not powered for subgroup analysis based on demographic characteristics surveyed, including age, gender, and race or ethnicity. We did note trends toward gender and racial differences in responses, with male students and White students reporting a greater increase in their sense of empowerment to address professionalism and safety issues compared to female and non-White respondents. These findings reflect existing literature on the learning environment and call attention to the need to address microaggressions and discrimination in the clinical learning environment. Students from groups underrepresented in medicine report less supportive and less positive learning environments.[34,40] Our finding of greater increase in empowerment in male and White students may reflect a more supportive learning environment for these students.

Our project has several limitations. An important limitation is the low response rate, particularly for the immediate postsurvey and 6-month postsurvey, despite electronic administration easily completed on handheld devices and multiple email reminders. The surveys were lengthy, which may have caused survey fatigue, contributing to the low response rate. Our small sample size limited power to assess differences based on characteristics such as gender, race, and ethnicity. Other limitations include several types of bias that may have occurred. The wording of the follow-up survey may have introduced recall bias. The high percentage of respondents who reported witnessing lapses in professional behavior (over 95% of respondents) may reflect a halo effect: Students who had witnessed lapses may have been more likely to complete the follow-up survey. We do not have details about the nature of professionalism lapses observed or which clerkships students had completed at the time of the 6-month survey. We are thus unable to determine the effect of specific clerkships on response rate, witnessed professionalism lapses, or speaking up. We queried satisfaction and self-assessment of learning, which are inherently limited by students' relative lack of experience and context in which to evaluate the curriculum. Lastly, we intended to gather data in the subsequent academic years; however, due to COVID-19 restrictions, the curriculum was not implemented. We thus report data from the 2 years of implementation.

Several changes may improve future iterations of this educational intervention. Students reported an increase in knowledge immediately after the exercise, and repeated exposure to similar curricula may be needed to effect behavior change or change the learning environment. Learners may benefit from repetitive practice of team communication skills, and subsequent simulations may reinforce these skills. The curriculum is adaptable to an online or remote synchronous platform using videoconferencing. This adaptation would require no significant changes to the material or the role-play exercise and could be employed for distance learning by institutions with learners at multiple sites.

This role-play simulation curriculum provides training and practice in interpersonal communication skills for medical students for use in the complex and often hierarchical patient care teams encountered in clerkships. Using a commonly encountered lapse in professionalism and focusing on patient safety, we demonstrated immediate improvement in knowledge and empowerment to engage in difficult conversations within a team.

Appendices

A. Case Summary.docx

B. Narrated Preclass Presentation.m4v

Copyright © 2023 Flier et al. This is an open-access publication distributed under the terms of the Creative Commons Attribution license.

C. Character Role Cards.docx

D. Flowchart for Simulation Role-Play.pdf

E. Brief and Debrief Guide.docx

F. Critical Actions Checklist.docx

G. SISS Pre- and Postsurveys.docx

All appendices are peer reviewed as integral parts of the Original Publication.

Lydia A. Flier, MD: Instructor, Department of Medicine, Mount Auburn Hospital and Harvard Medical School; ORCID: https://orcid.org/0000-0003-1165-8089

Jeremy B. Richards, MD, MA: Assistant Professor of Medicine, Harvard Medical School and Mount Auburn Hospital

Michele R. Hacker, ScD: Associate Professor, Department of Obstetrics and Gynecology, Beth Israel Deaconess Medical Center and Harvard Medical School; ORCID: https://orcid.org/0000-0003-0217-9991

Alexandra Hovaguimian, MD: Assistant Professor, Department of Neurology, Beth Israel Deaconess Medical Center and Harvard Medical School

Anita Vanka, MD: Assistant Professor, Department of Medicine, Beth Israel Deaconess Medical Center and Harvard Medical School; ORCID: https://orcid.org/0000-0001-6536-6925

Amy Sullivan, EdD: Director of Education Research, Shapiro Institute for Education and Research, Beth Israel Deaconess Medical Center and Harvard Medical School

Celeste S. Royce, MD: Assistant Professor, Department of Obstetrics, Gynecology and Reproductive Biology, Harvard Medical School, and Department of Obstetrics and Gynecology, Beth Israel Deaconess Medical Center; ORCID: https://orcid.org/0000-0002-0739-0174

Acknowledgments
The authors wish to acknowledge the assistance of Adrienne Erlinger, MPH, in the statistical analysis of this work and the assistance of Michael McBride, RN, and Darren Tavernelli, RN, from the Beth Israel Deaconess Medical Center Shapiro Simulation and Skills Center, in running the simulation sessions.

Disclosures
None to report.

Funding/Support
Dr. Hacker's work was conducted with support from Harvard Catalyst/the Harvard Clinical and Translational Science Center (National Center for Advancing Translational Sciences, National Institutes of Health Award UL 1TR002541) and financial contributions from Harvard University and its affiliated academic health care centers.

Dr. Royce received support from the Promising Young Investigator Grant, Department of Obstetrics and Gynecology, Beth Israel Deaconess Medical Center.

Simulation sessions occurred in and were supported by the Beth Israel Deaconess Medical Center Shapiro Simulation and Skills Center.

Prior Presentations
Flier LA, Hovaguimian A, Vanka A, Sullivan A, Royce CS. A professionalism curriculum for medical students entering clerkships. Poster presented at: Learn Serve Lead: The AAMC Annual Meeting; November 8-12, 2019; Phoenix, AZ.

Ethical Approval
The Harvard Medical School Office of Educational Quality Improvement deemed further review of this project not necessary.

References

1. Marcotte LM, Moriates C, Wolfson DB, Frankel RM. Professionalism as the bedrock of high-value care. *Acad Med*. 2020;95(6):864-867. https://doi.org/10.1097/ACM.0000000000002858
2. Krupat E, Dienstag JL, Padrino SL, et al. Do professionalism lapses in medical school predict problems in residency and clinical practice? *Acad Med*. 2020;95(6):888-895. https://doi.org/10.1097/ACM.0000000000003145
3. Martinez W, Lehmann LS, Thomas EJ, et al. Speaking up about traditional and professionalism-related patient safety threats: a national survey of interns and residents. *BMJ Qual Saf*. 2017; 26(11):869-880. https://doi.org/10.1136/bmjqs-2016-006284
4. Mak-van der Vossen M, Teherani A, van Mook WNKA, Croiset G, Kusurkar RA. Investigating US medical students' motivation to respond to lapses in professionalism. *Med Educ*. 2018;52(8): 838-850. https://doi.org/10.1111/medu.13617
5. Nasca TJ, Weiss KB, Bagian JP. Improving clinical learning environments for tomorrow's physicians. *N Engl J Med*. 2014; 370(11):991-993. https://doi.org/10.1056/NEJMp1314628
6. Wear D, Castellani B. The development of professionalism: curriculum matters. *Acad Med*. 2000;75(6):602-611. https://doi.org/10.1097/00001888-200006000-00009
7. Brennan N, Price T, Archer J, Brett J. Remediating professionalism lapses in medical students and doctors: a systematic review. *Med Educ*. 2020;54(3):196-204. https://doi.org/10.1111/medu.14016
8. Rougas S, Gentilesco B, Green E, Flores L. Twelve tips for addressing medical student and resident physician lapses in professionalism. *Med Teach*. 2015;37(10):901-907. https://doi.org/10.3109/0142159X.2014.1001730
9. Bradley F, Steven A, Ashcroft DM. The role of hidden curriculum in teaching pharmacy students about patient safety. *Am J Pharm Educ*. 2011;75(7):143. https://doi.org/10.5688/ajpe757143

Copyright © 2023 Flier et al. This is an open-access publication distributed under the terms of the Creative Commons Attribution license.

10. Shorey JM II. Signal versus noise on the wards: what "messages" from the hidden curriculum do medical students perceive to be importantly meaningful? *Trans Am Clin Climatol Assoc*. 2013; 124:36-45.

11. Rogers DA, Boehler ML, Roberts NK, Johnson V. Using the hidden curriculum to teach professionalism during the surgery clerkship. *J Surg Educ*. 2012;69(3):423-427. https://doi.org/10.1016/j.jsurg.2011.09.008

12. Kittmer T, Hoogenes J, Pemberton J, Cameron BH. Exploring the hidden curriculum: a qualitative analysis of clerks' reflections on professionalism in surgical clerkship. *Am J Surg*. 2013;205(4): 426-433. https://doi.org/10.1016/j.amjsurg.2012.12.001

13. Mak-van der Vossen MC, de la Croix A, Teherani A, van Mook WNKA, Croiset G, Kusurkar RA. A road map for attending to medical students' professionalism lapses. *Acad Med*. 2019;94(4): 570-578. https://doi.org/10.1097/ACM.0000000000002537

14. Ryan RM, Deci EL. Self-determination theory and the facilitation of intrinsic motivation, social development, and well-being. *Am Psychol*. 2000;55(1):68-78. https://doi.org/10.1037//0003-066X.55.1.68

15. Moyo M, Goodyear-Smith FA, Weller J, Robb G, Shulruf B. Healthcare practitioners' personal and professional values. *Adv Health Sci Educ Theory Pract*. 2016;21(2):257-286. https://doi.org/10.1007/s10459-015-9626-9

16. Green M, Zick A, Makoul G. Defining professionalism from the perspective of patients, physicians, and nurses. *Acad Med*. 2009;84(5):566-573. https://doi.org/10.1097/ACM.0b013e31819fb7ad

17. MM Al-Eraky. Twelve tips for teaching medical professionalism at all levels of medical education. *Med Teach*. 2015;37(11): 1018-1025. https://doi.org/10.3109/0142159X.2015.1020288

18. Hundert EM, Hafferty F, Christakis D. Characteristics of the informal curriculum and trainees' ethical choices. *Acad Med*. 1996;71(6):624-642. https://doi.org/10.1097/00001888-199606000-00014

19. Taylor DCM, Hamdy H. Adult learning theories: implications for learning and teaching in medical education: AMEE Guide no. 83. *Med Teach*. 2013;35(11):e1561-e1572. https://doi.org/10.3109/0142159X.2013.828153

20. Kirk LM. Professionalism in medicine: definitions and considerations for teaching. *Proc (Bayl Univ Med Cent)*. 2007; 20(1):13-16. https://doi.org/10.1080/08998280.2007.11928225

21. Hegazi I, Wilson I. Medical education and moral segmentation in medical students. *Med Educ*. 2013;47(10):1022-1028. https://doi.org/10.1111/medu.12252

22. Boenink AD, de Jonge P, Smal K, Oderwald A, van Tilburg W. The effects of teaching medical professionalism by means of vignettes: an exploratory study. *Med Teach*. 2005;27(5):429-432. https://doi.org/10.1080/01421590500069983

23. Bernabeo EC, Holmboe ES, Ross K, Chesluk B, Ginsburg S. The utility of vignettes to stimulate reflection on professionalism: theory and practice. *Adv Health Sci Educ Theory Pract*. 2013;18(3):463-484. https://doi.org/10.1007/s10459-012-9384-x

24. Aronson L. Twelve tips for teaching reflection at all levels of medical education. *Med Teach*. 2011;33(3):200-205. https://doi.org/10.3109/0142159X.2010.507714

25. Stern DT, Cohen JJ, Bruder A, Packer B, Sole A. Teaching humanism. *Perspect Biol Med*. 2008;51(4):495-507. https://doi.org/10.1353/pbm.0.0059

26. Rudolph JW, Simon R, Dufresne RL, Raemer DB. There's no such thing as "nonjudgmental" debriefing: a theory and method for debriefing with good judgment. *Simul Healthc*. 2006;1(1):49-55. https://doi.org/10.1097/01266021-200600110-00006

27. Diamond L, Izquierdo K, Canfield D, Matsoukas K, Gany F. A systematic review of the impact of patient–physician non-English language concordance on quality of care and outcomes. *J Gen Intern Med*. 2019;34(8):1591-1606. https://doi.org/10.1007/s11606-019-04847-5

28. Glod SA, Richard D, Gordon P, et al. A curriculum for clerkship students to foster professionalism through reflective practice and identity formation. *MedEdPORTAL*. 2016;12:10416. https://doi.org/10.15766/mep_2374-8265.10416

29. Strobel R, Antunez A, De La Rosa K, et al. Medical professionalism: a series of near-peer facilitated workshops for first-year medical students. *MedEdPORTAL*. 2017;13:10549. https://doi.org/10.15766/mep_2374-8265.10549

30. Lewis J, Allan S. Physician-patient boundaries: professionalism training using video vignettes. *MedEdPORTAL*. 2016;12:10412. https://doi.org/10.15766/mep_2374-8265.10412

31. Schwartzstein RM, Dienstag JL, King RW, et al; Pathways Writing Group. The Harvard Medical School Pathways curriculum: reimagining developmentally appropriate medical education for contemporary learners. *Acad Med*. 2020;95(11):1687-1695. https://doi.org/10.1097/ACM.0000000000003270

32. Riesenberg LA, Leitzsch J, Little BW. Systematic review of handoff mnemonics literature. *Am J Med Qual*. 2009;24(3): 196-204. https://doi.org/10.1177/1062860609332512

33. Baingana RK, Nakasujja N, Galukande M, Omona K, Mafigiri DK, Sewankambo NK. Learning health professionalism at Makerere University: an exploratory study amongst undergraduate students. *BMC Med Educ*. 2010;10:76. https://doi.org/10.1186/1472-6920-10-76

34. Chisholm LP, Jackson KR, Davidson HA, Churchwell AL, Fleming AE, Drolet BC. Evaluation of racial microaggressions experienced during medical school training and the effect on medical student education and burnout: a validation study. *J Natl Med Assoc*. 2021;113(3):310-314. https://doi.org/10.1016/j.jnma.2020.11.009

Copyright © 2023 Flier et al. This is an open-access publication distributed under the terms of the Creative Commons Attribution license.

35. Lucey C, Souba W. Perspective: the problem with the problem of professionalism. *Acad Med*. 2010;85(6):1018-1024. https://doi.org/10.1097/ACM.0b013e3181dbe51f
36. Bernard AW, Malone M, Kman NE, Caterino JM, Khandelwal S. Medical student professionalism narratives: a thematic analysis and interdisciplinary comparative investigation. *BMC Emerg Med*. 2011;11:11. https://doi.org/10.1186/1471-227X-11-11
37. Kenison TC, Madu A, Krupat E, Ticona L, Vargas IM, Green AR. Through the veil of language: exploring the hidden curriculum for the care of patients with limited English proficiency. *Acad Med*. 2017;92(1):92-100. https://doi.org/10.1097/ACM.0000000000001211
38. Burgess A, Bleasel J, Haq I, et al. Team-based learning (TBL) in the medical curriculum: better than PBL? *BMC Med Educ*. 2017;17:243. https://doi.org/10.1186/s12909-017-1068-z
39. Liaison Committee on Medical Education. *Functions and Structure of a Medical School: Standards for Accreditation of Medical Education Programs Leading to the MD Degree*. Association of American Medical Colleges/American Medical Association; 2021. Accessed October 13, 2023. https://lcme.org/wp-content/uploads/2022/07/2022-23_Functions-and-Structure_2022-04-01.docx
40. Orom H, Semalulu T, Underwood W III. The social and learning environments experienced by underrepresented minority medical students: a narrative review. *Acad Med*. 2013;88(11):1765-1777. https://doi.org/10.1097/ACM.0b013e3182a7a3af

Received: March 28, 2023
Accepted: August 18, 2023
Published: December 12, 2023

Copyright © 2023 Flier et al. This is an open-access publication distributed under the terms of the Creative Commons Attribution license.
